# Monitoring human exposure to four parabens and triclosan: comparing silicone wristbands with spot urine samples as predictors of internal dose

**DOI:** 10.1038/s41370-024-00663-0

**Published:** 2024-05-04

**Authors:** Jessica L. Levasseur, Kate Hoffman, Sharon Zhang, Ellen M. Cooper, Heather M. Stapleton

**Affiliations:** Nicholas School of Environment, Duke University, Durham, NC USA

**Keywords:** Personal exposure, Environmental monitoring, Parabens, Dermal exposure, Biomonitoring

## Abstract

**Background:**

People are exposed to a variety of chemicals each day as a result of their personal care product (PCP) use.

**Objective:**

This study was designed to determine if silicone wristbands provide a quantitative estimate of internal dose for phenols commonly associated with PCPs, with a focus on triclosan and four parabens: methyl-, ethyl-, propyl-, and butylparaben. Uptake of these compounds into wristbands and correlations with internal dose were assessed.

**Methods:**

Ten adults from central North Carolina wore five silicone wristbands, with one wristband removed each day for 5 days. Each participant provided a 24 h urine sample and a random spot urine sample each day, in which paraben and triclosan metabolites were evaluated.

**Results:**

All parabens and triclosan were detected frequently in wristbands and, except for butylparaben, in urine samples. Wristband and spot urine concentrations of parabens and triclosan were both compared to a measurement of internal dose (i.e., the total metabolite mass excreted over 5 days as a measurement of internal dose).

**Impact statement:**

The two most hydrophobic compounds investigated, butylparaben and triclosan, displayed significant linear uptake in wristbands over 5 days, whereas concentrations of methyl- and ethylparaben displayed a steady state concentration. In general, wristbands and spot urine samples were similarly correlated to internal dose for frequently detected parabens and triclosan.  However, wristbands have additional advantages including higher detection rates and reduced participant burden that may make them more suitable tools for assessing exposure to PCPs.

## Introduction

On average, Americans use nine personal care products (PCPs) per day, and through those products they are exposed to an estimated 126 chemicals [1]. There is increasing concern about the possible health implication of PCP use, particularly as exposure to PCP chemicals is thought to vary widely across sex and race/ethnicity, with some individuals exposed to many more PCP chemicals and at higher levels [2–7]. Additional studies evaluating exposure patterns and mixtures are needed to assess potential health impacts and vulnerable populations. However, measuring PCP exposure through traditional exposure assessment methods, such as urine biomonitoring, are invasive and can be burdensome for study participants. In addition, the short half-lives of some PCP chemicals in the body limits the utility of traditional biomonitoring approaches to understand chronic and time-variable exposure to PCP chemicals.

Silicone wristbands are a passive sampling tool that may overcome some of the obstacles associated with traditional biomonitoring methods used in exposure assessment. These exposure measurement devices accumulate chemicals from the ambient environment, with chemicals accumulating over the total time the wristband is worn. The utility of silicone wristbands in measuring human exposure to a wide range of semi-volatile organic compounds (SVOCs) [8–17] has been demonstrated in many studies [18–22]. Though silicone wristbands cannot capture dietary exposures, their ability to assess exposures to SVOCs in the ambient environment has been well documented for chemicals in which inhalation and dermal exposure routes dominate [8]. Exposure to some SVOCs is thought to be severely underestimated using traditional exposure assessment techniques, due to the consistent underestimation of dermal exposure [23, 24]. Silicone wristbands are thought to integrate both inhalation and dermal exposure routes [16] and therefore may provide an opportunity to better capture both dermal and personal cloud [25–27] exposures from PCPs over time. However, the utility of silicone wristbands as monitors of exposure to SVOCs found in PCPs remains unknown. A recent study demonstrated that a high frequency of lotion use among children was associated with elevated paraben levels in both silicone wristbands and urine and wristband paraben concentrations were positively associated with urinary paraben metabolite levels [10]. These results suggest that silicone wristbands may capture information about exposure to PCP chemicals, but further evaluation is needed.

In this study, we investigated whether silicone wristbands provide a quantitative estimate of internal dose for phenols often included as ingredients in PCPs [28–32], with a focus on methyl-, ethyl-, propyl-, and butylparaben, as well as triclosan. We evaluated how silicone wristbands and spot urine measurements of parabens and triclosan compare to internal dose, as calculated from 24 h urine samples. A second objective was to investigate the uptake rates of these chemicals on wristbands. Characterizing uptake rates on wristbands will allow for comparison across different wristband studies, including how the number of days of deployment matter for the values quantified on the wristband. Previous research has demonstrated that for some compounds, the uptake rate on wristbands is linear [33]. However, this past investigation focused on organophosphate esters (OPEs), a group of nonionic chemicals that are largely more hydrophobic than parabens, which are not associated with PCP use. Herein, we investigated the uptake rates for parabens and triclosan over the 5-day period to assess the reliability of wristbands as surrogates for internal dose.

## Materials and methods

### Study population

As described previously [33], a convenience sample of ten adults from North Carolina wore silicone wristbands and collect urine samples over 5 days (October 2018–June 2019). Participants started with five wristbands on a single wrist on day 1, removing one wristband each day thereafter. Participants were also asked to collect all voided urine over the full 5-day study period. Internal dose was calculated as the cumulative urinary metabolite mass excreted in urine over 5 days. In addition, one random spot urine sample was collected per person per day. These collections are discussed in more detail below. All study protocols were approved by the Duke University Institutional Review Board, and all participants provided written informed consent prior to participation in the study.

### Wristband collection

Commercially available silicone wristbands (24hourwristbands.com, Houston, TX, USA) were pre-cleaned through two 12 h Soxhlet extractions using first 1:1 ethyl acetate/hexane (v/v) and then 1:1 ethyl acetate/methanol (v/v) [17]. Wristbands were then dried in a vacuum oven at room temperature. When dried, wristbands were wrapped in precleaned aluminum foil (combusted at 450 °C) and stored in airtight jars until distributed to study participants.

Each participant began the study with five pre-cleaned wristbands on a single arm. Each day, participants removed one wristband, wrapped it in provided aluminum foil, and enclosed it in a plastic zip-top bag to be stored in their freezer. After 5 days, wristbands were transported to the research lab at Duke University and stored at −20 °C until analysis. In total, 49 wristbands were collected, as one study participant lost their day 5 wristband.

### Wristband extraction

Wristband extraction was based on a previous method in our laboratory [10], with a few modifications described herein (SI Table 1). The mass of a small piece (~0.75 g) of each wristband was first recorded and then cut into three pieces and transferred to a clean glass centrifuge tube for extraction. Wristbands were not rinsed prior to extraction, as we are interested in chemicals that accumulate via gaseous diffusion into the silicone and via interactions with particles that stick to the silicone [8, 16]. Samples were spiked with a suite of isotopically labeled internal standards and extracted using 1:1 hexane: acetone. Extracts were concentrated to 1.0 mL under nitrogen, then purified using a solid-phase extraction (SPE) Florisil cartridge (Sigma Aldrich Supelclean ENVI-Florisil SPE Tubes). Parabens and triclosan were eluted with 12 mL of ethyl acetate. These fractions were solvent exchanged to 1.0 mL methanol, transferred to autosampler vials, and filtered using 25 mm, 0.22 µm PTFE filters before analysis by liquid chromatography-electrospray ionization-tandem mass spectrometry (LC-ESI-MS/MS).

LC-MS/MS parameters were previously reported [10] with the following modifications: The mobile phases consisted of 0.8 mM ammonium acetate in (B) methanol and (A) LCMS water, and the flow rate was 0.4 mL/min. Initial conditions were 30% B held for 3 min, following a gradient to 95% B by 4 min and then increased to 98% B by 9 min, held at this ratio until 12.5 min before ramping down to 30% B from minutes 13 to 18. Further information on analysis parameters can be found in SI Table 2.

Isotopically labeled recovery standards were spiked into sample extracts prior to MS analysis and were used to calculate recovery of the internal standards. Recovery of the internal standards was assessed using ^13^C-Triclocarban (50 ng) for all internal standards (SI Table 3). Overall, lab processing blanks (solvent only, *n* = 4) and field blanks (unworn wristbands, *n* = 4) were analyzed alongside the wristband samples for quality assurance and control. Method detection limits (MDLs; ng/g) were determined by calculating three times the standard deviation of the average field blank levels for wristband samples and then dividing by the mass of the wristband [34].

### Urine collection

All participants were instructed to collect all urine excreted during the 5-day study in a provided kit that contained urine collection toilet hats, 24 h collection jugs, and spot sample tubes. All voids were pooled into a single 24 h jug each day. Participants were asked to also collect a spot sample from a randomly selected void each day by filling a 15 mL conical tube with urine. The remaining urine from that void was pooled with all other urine collected on that day to create a 24 h urine composite. Each participant’s spot urine samples and 24 h collection jugs were stored in their freezer or on ice for the duration of the study. Upon return to the research lab at Duke University, each sample’s volume was measured, urine was aliquoted, and samples were stored at −20 °C until analysis.

### Urine extraction

Specific gravity of each urine sample, pooled and spot, was measured through a refractometer (Atago) before extraction. Urine extraction was based on an adapted protocol from Ren et al. [35]. To extract the parabens and triclosan, urine samples (1 mL) were first incubated in a 37 °C water bath overnight after adding 1.75 mL of 1.0 M sodium acetate (pH 5) and 100 µL of a deconjugation enzyme solution (final concentration 1000 units/mL β-glucuronidase and 33 units/mL sulfatase activity). Internal standards were added before incubation (50 ng each of ^13^C methyl-, ethyl-, propyl-, and butylparabens, present as a mixed stock, and ^13^C triclosan). Empore C18SD SPE columns were used for solid phase extraction. Matrix spike test results ranged from 60 to 77% (SI Table 4). Information on the general recovery observed in these method development experiments are reported SI Table 5. For the urine analyses, parabens and triclosan were extracted using Empore C18SD SPE tubes (10 mm/6 mL cartridges) using 8 mL methanol before being concentrated under nitrogen. Final extracts were spiked with 50 ng each of the recovery standards (D_4_- methyl- and propylparaben, present as a mixed stock, and D_3_-triclosan) and then filtered through a 0.2 µm PTFE membrane filter (25 mm) before being analyzed using LC-MS/MS.

Instrument settings were similar to those described above for wristbands. The mobile phases consisted of 0.8 mM ammonium acetate in (A) methanol and (B) LCMS-water, and the flow rate was 0.5 mL/min. The same LC-MS conditions were used as described above for wristbands. Recovery of the internal standards was assessed using D_3_-triclosan (50 ng) and D_4_-methyl- and propylparaben (50 ng). Four urine Standard Reference Material (SRM 3673, NIST, Gaithersburg, MD) samples (1 mL each) were analyzed alongside samples (SI Table 5), as well as six duplicate samples, and ten lab blanks for quality assurance and quality controls. MDLs were calculated as described for wristbands. Internal standards’ recovery of isotopically labeled methyl-, ethyl-, propyl-, and butylparaben ranged from 61 to 88%, and recovery for triclosan was 73%.

### Statistical analysis

In total, 49 wristbands and 100 urine samples were analyzed for parabens and triclosan. Detection frequencies and data distributions were assessed. Statistical analyses were performed for all compounds with detection frequencies ≥85%. For these compounds, chemical concentrations that fell below the MDL were replaced by MDL divided by the square root of 2 for statistical analyses. Concentrations of parabens and triclosan in urine samples were specific gravity corrected, to account for dilution [36], and wristbands were corrected for the weight of the sample analyzed to report concentrations in ng of chemical per gram of wristband. Preliminary analyses suggested skewed chemical concentrations in wristbands and urine samples. Accordingly, non-parametric analyses methods were used, or concentrations were log10 transformed to improve normality before analyses were conducted. Statistical analyses were performed using SAS statistical software (version 9.4; SAS Institute Inc., Cary, NC) and in R Studio (version 2022.07.2-576, R Studio, Boston, MA). All results were assessed at *α* = 0.05 for statistical significance.

Daily uptake in wristbands was calculated using a regression equation based on the geometric mean of each chemical measured per day, across all participants. Spearman correlations were used to assess multiple relationships: correlations between wristbands and internal dose, random spot urine and internal dose, and each day’s spot urine and internal dose. Typically, exposure studies of parabens and triclosan have collected a single spot urine sample. However, our study design included daily spot urine samples. To assess how well randomly selected spot urine samples compared to the internal dose (similar to the information typically available in biomonitoring studies), we randomly selected 1 spot urine sample from each participant and evaluated correlations with total exposure over 5 days. This procedure was repeated 1000 times, to acquire a distribution of correlations between random spot urine and total mass excreted (R version 4.3.1). These random spot urine samples were assessed for Spearman correlations against internal dose. To assess the reproducibility of spot urine samples, interclass correlation coefficients (ICC) were calculated for spot urine samples over 5 days. ICCs are the ratio of between-subject variability to total variability across a population and range from 0 to 1. Poor reproducibility is interpreted from an ICC below 0.4, moderate to good reproducibility is assessed for ICCs ranging from 0.4 to 0.7, and ICCs above 0.7 are considered to have excellent reproducibility [37].

## Results

### Parabens and triclosan in silicone wristbands

Parabens and triclosan were detected frequently in wristbands worn by study participants on all 5 days. By day 3, all compounds were measured in every wristband (Table 1). Among the parabens, those most commonly reported in PCPs (methyl-, ethyl-, and propylparaben) [38] were found in the highest median amounts on the day 5 wristbands (6.15, 1.64, and 11.0 ng/g, respectively), though triclosan was found at the second highest median concentration overall (9.82 ng/g).

**Table 1:** Descriptive statistics for parabens and triclosan measured in silicone wristbands.

Compound	MDL (ng/g)	Number of bands > MDL					Day 5 Statistics^a^			Daily Uptake (ng/g/day)	Daily Uptake *p* value^b^
		Day 1 *N* = 10	Day 2 *N* = 10	Day 3 *N* = 10	Day 4 *N* = 10	Day 5 *N* = 9	Minimum (ng/g)	Median (ng/g)	Maximum (ng/g)		
Methylparaben	0.50	10	10	10	10	9	3.97	6.15	252	0.16	0.95
Ethylparaben	0.23	8	10	10	10	9	0.47	1.64	431	0.39	0.55
Propylparaben	1.45	6	9	10	10	9	4.09	11.0	846	3.5	0.38
Butylparaben	0.07	6	10	10	10	9	0.22	0.65	1.32	0.12	**0.01**
Triclosan	0.54	10	10	10	10	9	5.67	9.82	316	3.6	**0.03**

^a^Day 5 statistics *N* = 10 because one participant’s day 4 wristband was used as their day 5 wristband because they lost their day 5 wristband.

^b^Bolded values are significant at *p* < 0.05.

### Urinary biomarkers of paraben and triclosan exposure

The parabens that are most commonly reported as ingredients in PCPs (methyl-, ethyl-, and propylparaben) [38] were detected frequently in both spot and pooled urine samples (Table 2). Butylparaben was detected less frequently, consistent with other studies [10, 39–41], and was not included in subsequent statistical analyses. Spot urine sample medians ranged from 0.18 ng/mL (propylparaben) to 1.89 ng/mL (methylparaben), following a similar pattern of abundance in wristbands. Methyl- and propylparaben had the highest maximums in spot urine samples of the compounds measured herein, with methylparaben reporting a maximum of 152 ng/mL and propylparaben reporting a median of 128 ng/mL. Pooled urine concentration values ranged from a median of 0.18 ng/mL (triclosan) to 2.74 ng/mL (methylparaben), and maximum ranged from 3.91 ng/mL (triclosan) to 183 ng/mL (methylparaben), with methyl- and propylparaben again reporting the highest maximums of the compounds measured (propylparaben = 93.0 ng/mL).

**Table 2:** Descriptive statistics for urinary paraben and triclosan metabolite results (ng/mL), corrected for specific gravity.

Compounds	MDL (ng/mL)	Spot Samples (*n* = 50), ng/mL				Spot ICC	Daily Pools (*n* = 50), ng/mL			
		% Detect	Minimum	Median	Maximum		% Detect	Minimum	Median	Maximum
Methylparaben	0.56	98	<MDL	1.89	152	0.62 (0.36–0.89)	100	1.00	1.62	77
Ethylparaben	0.03	96	<MDL	0.77	46.0	0.69 (0.46–0.93)	98	<MDL	0.38	26.1
Propylparaben	0.06	85	<MDL	0.18	128	0.36 (0.04–0.68)	85	<MDL	0.11	46.3
Butylparaben	0.11	9	<MDL	<MDL	0.43	--	4	<MDL	<MDL	0.19
Triclosan	0.03	94	<MDL	0.21	3.32	0.80 (0.64–0.97)	98	<MDL	0.08	2.20

### Comparison of urine and wristband measurements in assessing exposure to chemicals found in personal care products

**Fig. 1:**
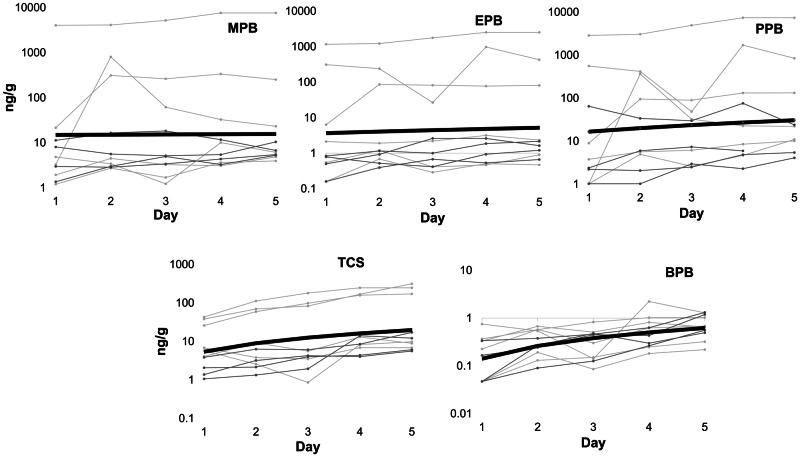
Paraben and triclosan concentrations in day 5 silicone wristbands (left, black) and median modeled spot urine samples with total range of spearman correlations shown below(*n* = 1000) (right, blue) as predictors of total paraben or triclosan metabolite excreted in 5 days (internal dose). Spearman correlation coefficients are reported along with *p* values (with bolded values significant at *p* < 0.05) in correlation plots for wristbands versus internal dose. Median Spearman correlations shown for random spot urine values versus internal dose are bolded if significant at *p* < 0.05. Log scales are provided on both the x and y axis in all graphs.

Randomly chosen spot urine samples were assessed in this study to approximate biomonitoring sample collection commonly employed in epidemiology studies. The ten participants, each with five spot urine samples, allowed for many combinations of one random spot urine sample to be chosen per person. These differences in the spot urine sample chosen can lead to differences in correlations between a random spot urine sample and total mass excreted over the duration of the study. To address the variation in correlations that may be associated with a random set of spot urine samples as compared to total mass excreted in urine, 1000 permutations of random spot urine samples were created in R. This code randomly selected one spot urine per person, 1000 times. Spearman correlations were conducted on these 1000 permutations of random spot urine data as compared to internal dose. The number of random permutations that were significant at *p* < 0.05 varied per compound: only 24% of permutations for methylparaben, 86% of permutations for ethylparaben, and 59% of permutations for propylparaben, while 100% of permutations for triclosan were significant at *p* < 0.05. For the full set of 1000 permutations, Spearman correlation ranges were relatively large: methylparaben (*r*_*s*_ = 0.15–0.88), ethylparaben (*r*_*s*_ = 0.37–0.94), propylparaben (*r*_*s*_ = 0.15–0.96), and triclosan (*r*_*s*_ = 0.64–0.99). This was compared with a correlation analysis between the concentrations of each chemical in day 5 wristbands versus total internal dose. Results and median Spearman correlations per compound are shown in Fig. 1.

## Discussion

### Parabens and triclosan in silicone wristbands

Compared to previous measurements in silicone wristbands, paraben and triclosan levels were lower in this study than in our previous assessment of children in North Carolina, though in our previous study wristbands were worn by children for 7 days (compared to 5 days by adults herein). Patterns of exposure were similar between studies in that triclosan and propylparaben were measured in the highest amounts in both our current and prior work [10]. Differences in exposure and behavior between children (aged 3–6) and adults may explain this pattern, as may the difference in duration of time the wristbands were deployed. In addition, these studies used different silicone wristbands (i.e., children’s bands were size-adjustable and purchased from another manufacturer). Further, our previous study was conducted from 2014 to 2016 [10], before the September 2017 ban on triclosan in over-the-counter antimicrobial ingredients was instituted by the United States Food and Drug Administration [42]. To our knowledge, no other studies using silicone wristbands have measured parabens. Only a few studies to date [10, 43, 44] have measured triclosan in wristbands. Results here are similar to our previous research for both parabens and triclosan [10]. However, triclosan detection frequencies differed from those reported by Romano et al., where only an 8% detection frequency for triclosan was reported (parabens not measured) [43, 44]. These triclosan detection frequency differences are likely due to differences in the analytical methods employed that resulted in different method detection limits.

**Fig. 2:**
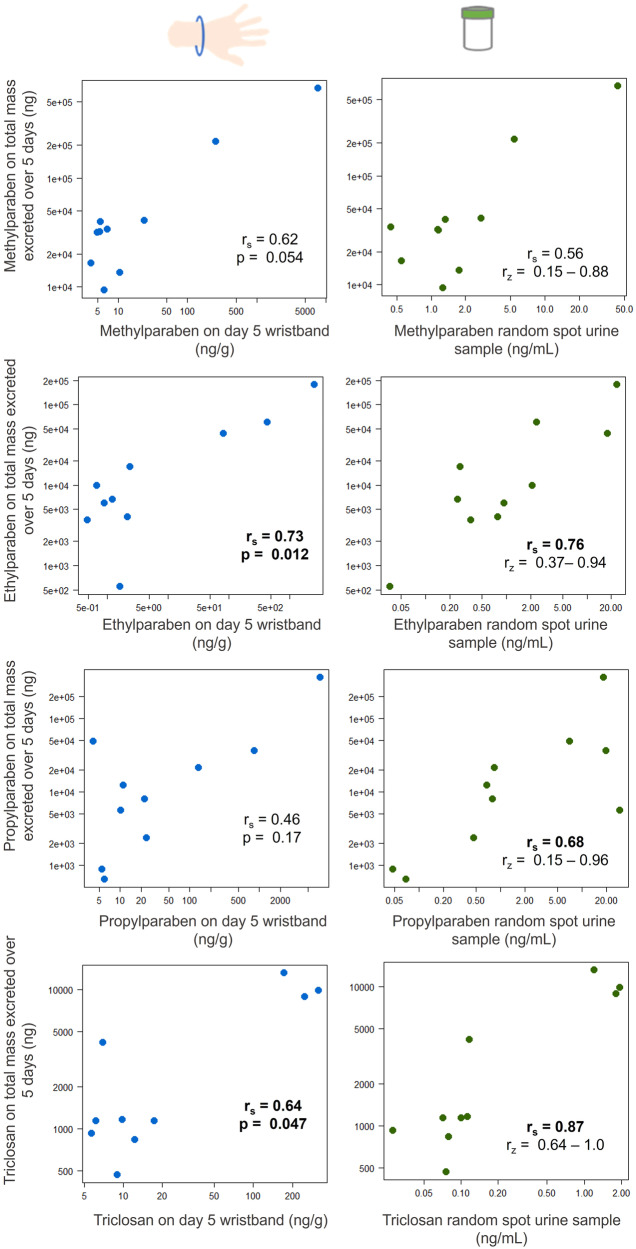
Wristband measurements of four parabens (methyl-, ethyl-, propyl-, and butylparaben, labeled as MPB, EPB, PPB, and BPB, respectively) and triclosan (TCS) (ng/day) over 5 days are shown here. All 10 participants are represented with a different color. The thick black line represents the geometric mean uptake. Geometric mean uptake for both butylparaben and triclosan was significant at *p* < 0.05.

Table 1 shows the daily uptake rates in wristbands for each chemical investigated. Uptake of triclosan was linear over time in wristbands (uptake rate = 3.6 ng/g/day; *p* = 0.03), as was uptake of butylparaben (uptake rate = 0.12 ng/g/day; *p* = 0.01). The lack of significance for the other compounds may be that these smaller, more water-soluble compounds reached an equilibrium, or steady-state, concentration more quickly than the other analytes. Log *K*_*ow*_ values for these compounds range from 1.96 (methylparaben) to 4.97 (triclosan). Triclosan and butylparaben are the most hydrophobic of this group (butylparaben Log *K*_*ow*_ 3.57) and methyl-, ethyl-, and propylparaben are more hydrophilic. The lack of statistical significance in uptake rates for the parabens with lower Log *K*_*ow*_ values may be due to a “washing off” (e.g., from showering/bathing). A visual demonstration of the uptake over time, for each study participant and the average daily uptake, can be found in Fig. 2. Better understanding of how uptake in wristbands can be influenced by deployment is essential in understanding how different silicone wristband studies may be compared. If, as is the case with butylparaben and triclosan, uptake is linear, we can easily compare across studies with different durations of wristband deployment. If uptake is non-linear, such as for methyl-, ethyl-, and propylparaben, a chemical may be reaching equilibrium in the wristbands, and comparisons across studies with different deployment times should be done with caution.

### Urinary biomarkers of paraben and triclosan exposure

Our results or consistent with prior studies of paraben exposure despite some key differences in study design. For example, other studies that measured parabens in urine have primarily focused on women, and particularly pregnant women [39, 41, 45–48], while our study included both women and men. One study that looked exclusively at men reported higher concentrations of parabens in urine than our study, though the relative ranking of concentrations was conserved (methylparaben having the highest concentration, followed by propyl- and butylparaben; ethylparaben was not measured) [40]. Another study that reported exclusively on children’s urinary concentration of parabens and triclosan found a similar pattern for paraben concentrations (highest to lowest concentration: methyl-, propyl-, ethyl-, and butylparaben) as this investigation, though triclosan concentrations in urine were much higher than observed here [10, 19]. This may be due to the increased hand-to-mouth behavior of children as compared to adults, as that same study measured relatively high levels of triclosan in house dust [10], or due to the fact that triclosan was banned by the U.S. Food and Drug Administration in September 2016 [49]. Due to the small sample size of this study, it is also possible that other behaviors may have been different between our study population and previous studies, which would influence the differences noted herein.

It is generally accepted that parabens have a short half-life in the body, estimated at less than 24 h. Triclosan also has a short half-life of ~21 h [50]. We assessed the relative variability within an individual compared to the variability in our study sample by calculating ICCs for spot urine samples. The observed ICCs ranged from 0.36 (95% CI: 0.04–0.68) for propylparaben to 0.80 (95% CI: 0.64–0.97) for triclosan, the largest and most hydrophobic of the compounds investigated. ICCs calculated herein (Table 2) were within the range of those previously reported in the literature (reported values for methylparaben: 0.24–0.87; ethylparaben: 0.40–0.89; propylparaben: 0.26–0.78; triclosan 0.59–0.99) [39–41, 45, 46, 48, 51–53]. Interestingly, variation in the timing of sample collection (multiple urine collections ranging from 2 days apart to 1273 days apart) across these other studies and our small sample size (*n* = 10 adults) did not contribute to large, observed differences.

### Comparison of urine and wristband measurements in assessing exposure to chemicals found in personal care products

A central objective of this study was to determine if a wristband measurement of parabens and triclosan predicts the total internal dose (approximated here using the total mass of a compound excreted in urine over 5 days). To address this, we first investigated how strongly each exposure measurement matrix (spot urine and silicone wristbands), correlated to total internal dose. All four compounds were positively correlated between day 5 wristbands and internal dose, with statistical significance observed for ethylparaben (*r*_*s*_ = 0.73, *p* = 0.012) and triclosan (*r*_*s*_ = 0.64, *p* = 0.047) and near statistical significance for methylparaben (*r*_*s*_ = 0.62, *p* = 0.054). In our simulation using randomly selected sport urine samples, spearman correlations with internal dose ranged from 0.56 (methylparaben) to 0.87 (triclosan), but significance varied per compound across the 1000 simulations, as discussed above. Wristband Spearman correlations were well within the ranges of random spot urine versus internal dose (listed above), each tending to the higher end of the Spearman correlation ranges for the 1000 permutations of random spot urine samples. While the magnitude of the correlation coefficients was similar between the two (wristbands versus internal dose and median modeled correlation of random spot urine versus internal dose), significance for random spot urine samples varied widely across the simulation. It is important to note that these estimates result from data collected from only ten individuals, and there is some uncertainty in these estimates. However, these results imply that wristbands may perform better than random spot urine samples in predicting internal dose for parabens and triclosan.

Our results should be interpreted in the context of several important limitations. Despite many samples (100 urine samples and 49 wristbands), our study had a relatively small number of participants. Our samples size may have limited our ability to detect important statistical trends and our study population may not be representative of the general population. Further investigations of bathing or handwashing as well as temperature would be helpful in informing future research, as this has not yet been investigated. Impacts of these hygienic practices may influence wristband loading of particular compounds based on their physical-chemical properties [8]. Nonetheless, this is the first study of its kind to measure how well wristbands measure chemicals commonly found in PCPs as compared to traditionally used spot urine samples and compare them back to “internal dose.” Ideally, future investigations should be conducted with larger populations and should consider collecting information on individual behavior, particularly the use of PCPs and hygiene habits.

## Conclusion

Previous research has observed good correlation between wristband measurements of phenols and traditional biomarkers of exposure, but how well these measures predict total exposure over time has been unknown [10]. In this study, we investigated whether wristbands or spot urine better predicted total exposure to parabens and triclosan over a 5 day period. This study demonstrates that wristbands perform similarly to spot urine at predicting internal dose over 5 days for three parabens (methyl-, ethyl-, and propylparaben) and triclosan. When measuring all five compounds, wristbands appeared to have an increased ability to detect exposure as compared to spot urine or pooled urine samples, as wristbands had 100% detection frequency for all compounds by day 3 (Table 1). In comparison, the detection frequency for urinary measurements ranged from 85 to 100% for methyl-, ethyl-, propylparaben and triclosan, and measurements of butylparaben were too low for statistical analysis (spot urine = 9% detection frequency, daily pooled urine = 4% detection frequency; Table 2). Overall, this study concludes that wristbands can provide a quantitative assessment of exposure and internal dose of compounds associated with PCPs such as parabens and triclosan, particularly as compared to traditional spot urine assessments.

The two most hydrophobic compounds investigated, butylparaben and triclosan, had the highest linear uptake in wristbands of the compounds examined here. Though lower than the linear uptake rates quantified for OPEs [33], determining linear uptake rates in wristbands may allow for better understanding of how wristband deployment differences may influence quantification of chemicals on wristbands. Even if uptake in wristbands is not linear, this investigation sheds light on what may drive uptake of particular chemicals, including investigating if some chemicals may reach an equilibrium. Knowing the uptake rate per compound in wristbands will allow us to better compare across studies of chemical exposure using silicone wristbands and will allow better understanding of how uptake and deployment matter in silicone wristband studies.

Further, the levels of triclosan measured herein imply there is consistent exposure to triclosan, despite its 2016 phase out, consistent with earlier findings [10, 54]. Other possible sources of exposure to triclosan must be identified to address this ongoing exposure.

Ultimately, though wristbands and spot urine samples perform similarly at predicting total exposure to some parabens and triclosan over 5 days, wristbands may have improved utility as an exposure measurement matrix. It is worth considering the benefits of sample collection via wristbands as compared to urinary biomonitoring. Wristbands are less invasive, easier to handle for participants and analysis centers (i.e., they can be mailed back and forth to participants), and less burdensome for study participants than collecting urinary samples. Wristbands can be used to quantify a large swath of SVOCs at once, which may have implications in their utility investigating exposure to real-world mixtures of chemicals such as those found in PCPs. Wristbands may also have promise as exposure measurement devices that may better reach marginalized populations that often experience disproportionate exposures. Given that chemical exposures often vary by race due to factors such as place [2, 55] and differences in product use [3–7], wristbands may be an affordable, non-invasive way to monitor and/or measure these exposure disparities more effectively that typical biomonitoring methods in underserved populations.

### Supplementary information

Supplementary Information

## Data Availability

The de-identified datasets generated during and/or analyzed during the current study are available from the corresponding author upon request.
