# Supplementary material for: Monitoring human exposure to four parabens and triclosan: comparing silicone wristbands with spot urine samples as predictors of internal dose
Source: J Expo Sci Environ Epidemiol. 2024 May 4;34(4):670–8. doi: 10.1038/s41370-024-00663-0 (PMC11303247; doi:10.1038/s41370-024-00663-0)

**Supplemental Information**

SI Table 1. Comparison of paraben recovery in wristband methods, comparing the methods used by Levasseur et al., 2021 to this study.

SI Table 2. m/z for four parabens and triclosan

SI Table 3. Percent recovery of parabens and triclosan measured in all samples and blanks (n = 10) for silicone wristbands

SI Table 4. Matrix spike test for urine extraction method; 50 ng of each isotopically labelled internal standard used to calculate percent recovery in the matrix spike tests.

SI Table 5. Levels of urinary metabolites measured in lab processing blanks (n = 10) and the SRM 3673 NIST (n = 4).

SI Table 1: Comparison of paraben recovery in wristband methods, comparing the methods used by Levasseur et al., 2021 to this study.


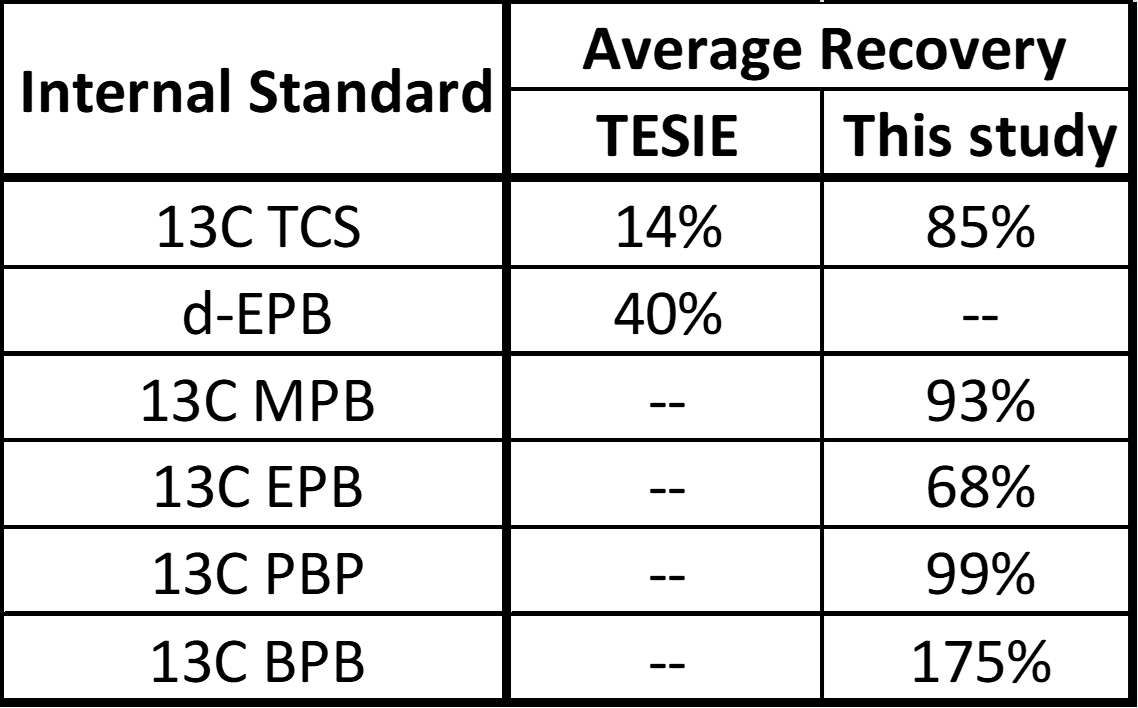


SI Table 2: m/z for four parabens and triclosan


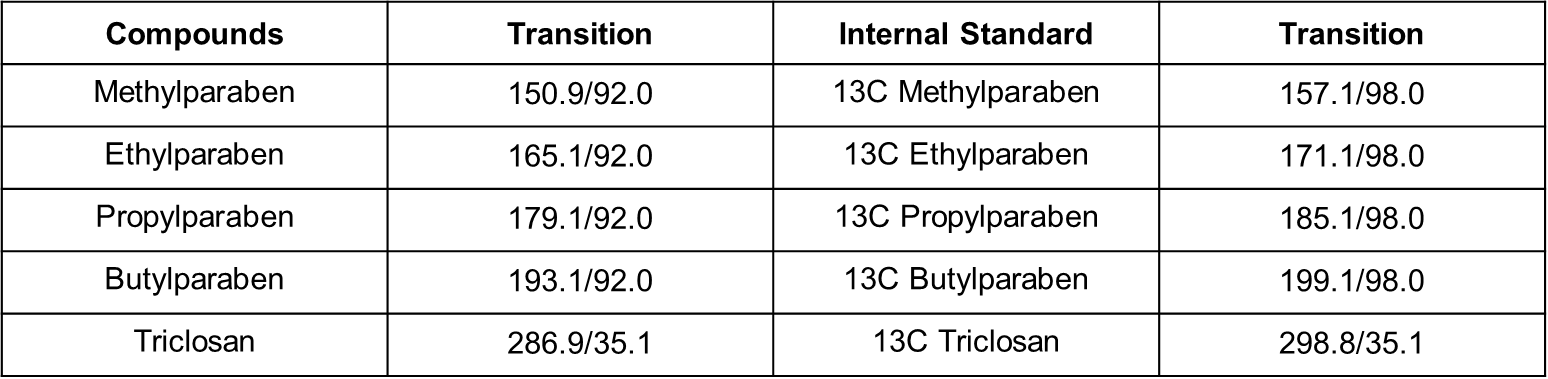


SI Table 3: Percent recovery of parabens and triclosan measured in all samples and blanks (n = 10) for silicone wristbands


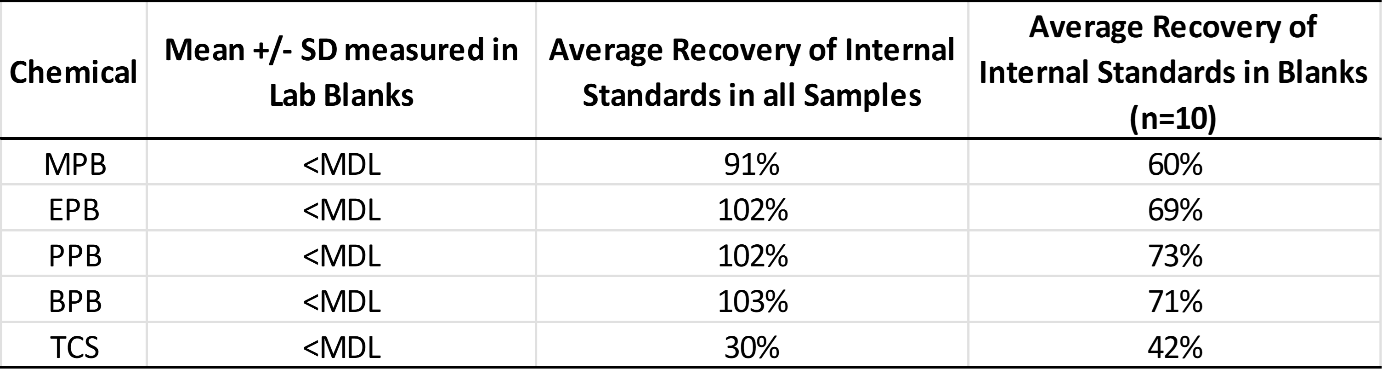


SI Table 4. Matrix spike test for urine extraction method; 50 ng of each isotopically labelled internal standard was used to calculate percent recovery in the matrix spike tests.


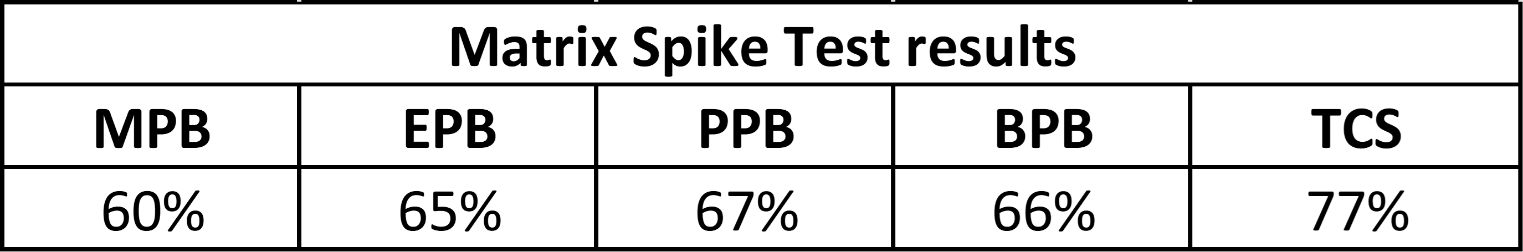


SI Table 5: Levels of urinary metabolites measured in lab processing blanks (n = 10) and the SRM 3673NIST (n = 4).


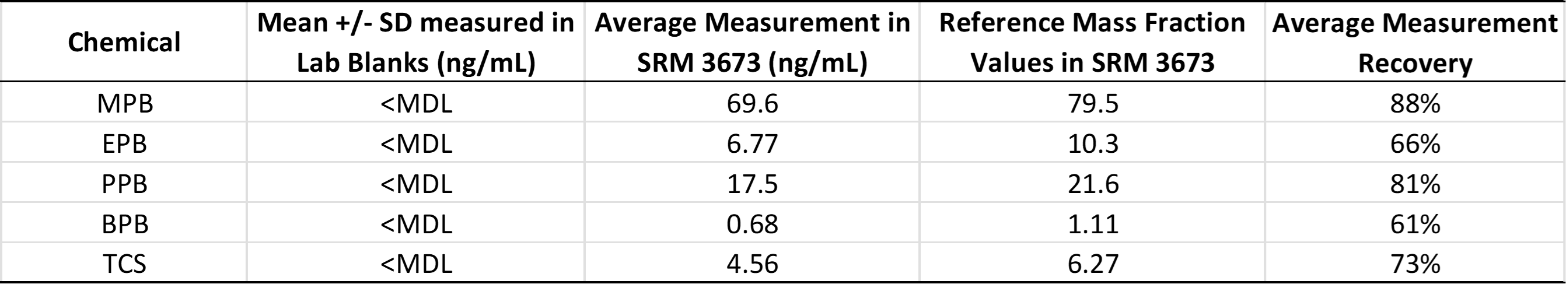

Supplement: Supplementary file 1 — Supplementary Information [file 41370_2024_663_MOESM1_ESM.docx]
